# Rapid and simple detection of *Phytophthora cactorum* in strawberry using a coupled recombinase polymerase amplification–lateral flow strip assay

**DOI:** 10.1186/s42483-021-00089-8

**Published:** 2021-06-10

**Authors:** Xinyu Lu, Heng Xu, Wen Song, Zitong Yang, Jia Yu, Yuee Tian, Min Jiang, Danyu Shen, Daolong Dou

**Affiliations:** 1 College of Plant Protection, Nanjing Agricultural University, Nanjing, 210095 China; 2 Department of Plant Protection, Henan University of Science and Technology, Luoyang, 471000 China; 3 Pingyi County Forestry Development Center, Linyi, 273300 China

**Keywords:** Alkaline lysis extraction, Lateral flow assay, *Phytophthora cactorum*, Rapid diagnosis, Recombinase polymerase amplification

## Abstract

*Phytophthora cactorum* is a devastating pathogen that infects a wide range of plants and causes Phytophthora rot disease, which has resulted in great economic losses in crop production. Therefore, the rapid and practicable detection of *P. cactorum* is important for disease monitoring and forecasting. In this study, we developed a lateral flow recombinase polymerase amplification (LF-RPA) assay for the sensitive visual detection of *P. cactorum*. Specific primers for *P. cactorum* were designed based on the ras-related protein gene *Ypt1*; all 10 *P. cactorum* isolates yielded positive detection results, whereas no cross-reaction occurred in related oomycete or fungal species. The detection limit for the LF-RPA assay was 100 fg of genomic DNA under optimized conditions. Combined with a simplified alkaline lysis method for plant DNA extraction, the LF-RPA assay successfully detected *P. cactorum* in naturally diseased strawberry samples without specialized equipment within 40 min. Thus, the LF-RPA assay developed in this study is a rapid, simple, and accurate method for the detection of *P. cactorum*, with the potential for further application in resource-limited laboratories.

**Supplementary Information:**

The online version contains supplementary material available at 10.1186/s42483-021-00089-8.

## Background

Strawberry is a nutritive and lucrative fruit crop that is commonly grown in temperate areas worldwide. China is one of the largest strawberry-producing countries; the Chinese strawberry industry has grown steadily in recent years, occupying an important position among Chinese economic crops. Pathogen infection is the main problem affecting strawberry yield and post-harvest quality (Okayama 1993; Barbey et al. 2019). The major pathogen genera threatening strawberry development include *Phytophthora*, *Botrytis*, *Xanthomonas*, *Colletotrichum*, and *Aspergillus*. Due to their damaging effects, few nurseries produce certified disease-free plants.

Phytophthora crown rot is among the most destructive diseases in strawberry production, limiting fruit harvest and causing considerable economic losses. This disease is caused by *Phytophthora cactorum*, an oomycete species that causes damage in both agricultural production and natural ecosystems (de Andrade Lourenco et al. 2020). *P. cactorum* has a wide host range, infecting around 160 herbaceous and woody plants (Li et al. 2013). Strawberry plants infected with *P. cactorum* usually show wilting and stunting, with leather rot or softening symptoms in fruits. Controlling *P. cactorum* disease has been hampered by its rapid spread and long-term survival in soil. Therefore, the rapid and accurate detection of *P. cactorum* during the early infection stages is critical for disease management.

The traditional plant pathogen detection method of isolation, culture, and pathogenicity testing according to Koch’s postulates is time consuming and requires professional training. Other methods of plant pathogen detection include monoclonal antibody testing, enzyme-linked immunosorbent assays, and polymerase chain reaction (PCR) analysis (Levesque 2001). However, these techniques require expensive materials and equipment that may not be available in resource-poor regions. Therefore, the development of a convenient pathogen detection method is important for sustainable agricultural production. Among molecular detection methods, loop-mediated isothermal amplification (LAMP) and recombinase polymerase amplification (RPA) are simple and effective detection approaches. DNA amplification and product detection via LAMP are achieved under isothermal conditions using *Bst* polymerase. The reaction is incubated at 60–65 °C for about 60 min, normally requiring four to six primers to increase specificity. Compared with LAMP, RPA has no strict requirements for template integrality, reaction temperature, or reaction time, and therefore has the potential for application in portable nucleic acid detection.

RPA is an ideal candidate as a fast, reliable, and portable diagnostic assay, especially under resource-limited circumstances. Many studies have shown that RPA can be performed at 25–45 °C without the use of complicated equipment, which overcomes environmental constraints. These advantages have allowed RPA to be used successfully in the detection of various pathogens such as viruses (Hou et al. 2017; Tu et al. 2017; Yang et al. 2017; Miao et al. 2019), bacteria (Gao et al. 2018; Zhang et al. 2019), and parasites (Castellanos-Gonzalez et al. 2018; Hassan et al. 2018; Wu et al. 2019). Although RPA has been frequently applied to detect pathogens related to humans and domestic animals, its application in plant pathogen detection remains poorly studied. Recently, isothermal RPA assays for the detection of *Phytophthora* pathogens were successfully established (Dai et al. 2019a; Yu et al. 2019; Dai et al. 2020; Lu et al. 2020).

In this study, we developed a rapid and simple lateral flow (LF)-RPA assay for the detection of *P. cactorum* by targeting the ras-related protein gene *Ypt1* of *P. cactorum*. We optimized the amplification temperature and detection time of the LF-RPA assay. We also compared the detection results with those of traditional PCR using 10-fold serial dilution of *P. cactorum* genomic DNA and crude extracts from infected strawberry as templates. The LF-RPA assay enabled the rapid and simple detection of *P. cactorum* in resource-limited laboratories.

## Results

### Screening of specific primers

We designed three RPA primer pairs based on specific regions of the *Ypt1* gene of *P. cactorum* by comparing *Ypt1* sequences derived from *P. cactorum* and closely related *Phytophthora* species (Additional file 1: Table S1). Initially, we performed a conventional PCR assay using *P. cactorum* genomic DNA as a template to evaluate these primer pairs based on sensitivity tests. The detection limit for Pcac-F2/R2 and Pcac-F3/R3 was 1 pg, which was more sensitive than that for Pcac-F1/R1 (Additional file 2: Figure S1a). For further specificity evaluation of the Pcac-F2/R2 and Pcac-F3/R3 primers using conventional PCR, reactions were performed using genomic DNA from 59 isolates as templates, including 10 *P. cactorum* isolates and 18 isolates from 10 other *Phytophthora* species, 16 *Pythium* species, and 4 fungal species. The target products were clearly observed in only the 10 *P. cactorum* isolates; no amplification bands were visualized in the other *Phytophthora*, *Pythium*, or fungal isolates (Additional file 2: Figure S1b and Table 1). Notably, there was no cross-reactivity between *P. cactorum* and closely related species such as *P. infestans*, *P. ipomoeae*, *P. mirabilis*, or *P. parasitica* (Additional file 2: Figure S1b). Therefore, the Pcac-F2/R2 and Pcac-F3/R3 primers were highly specific for *P. cactorum*. We randomly selected Pcac-F3/R3 for the following RPA assay, and the corresponding probe PcacProb was accordingly designed. The specificity of Pcac-F3/R3 was also confirmed by the LF-RPA assay (Table 1).

**Table 1:** Isolates used for specificity test of the LF-RPA assay

Species	Number of isolates	Clade	LF-RPA
*Phytophthora cactorum*	10	1	+
*Phytophthora infestans*	5	1	–
*Phytophthora ipomoeae*	2	1	–
*Phytophthora mirabilis*	2	1	–
*Phytophthora parasitica*	3	1	–
*Phytophthora palmivora*	1	4	–
*Phytophthora capsici*	1	2	–
*Phytophthora sojae*	1	7	–
*Phytophthora megasperma*	1	6	–
*Phytophthora cryptogea*	1	8	–
*Phytophthora drechsleri*	1	8	–
*Pythium spinosum*	1	/	–
*Pythium intermedium*	1	/	–
*Pythium helicoides*	1	/	–
*Pythium ultimum*	1	*/*	*–*
*Pythium aphanidermatum*	1	*/*	*–*
*Pythium irregulare*	1	*/*	*–*
*Pythium arrhenomanes*	1	*/*	*–*
*Pythium hydnosporum*	1	*/*	*–*
*Pythium marsipium*	1	*/*	*–*
*Pythium dissotocum*	1	*/*	*–*
*Pythium catenulatum*	1	*/*	*–*
*Pythium splendens*	1	*/*	*–*
*Pythium heterothallicum*	1	*/*	*–*
*Pythium sylvaticum*	1	*/*	*–*
*Pythium oligandrum*	1	*/*	*–*
*Pythium periplocum*	1	*/*	*–*
*Fusarium graminearum*	1	*/*	*–*
*Sclerotinia sclerotiorum*	1	*/*	*–*
*Botrytis cinerea*	1	*/*	*–*
*Rhizopus oryzae*	1	*/*	*–*

^“^+”, positive amplification; “-”, negative amplification

### Optimal conditions for the LF-RPA assay

**Fig. 1:**
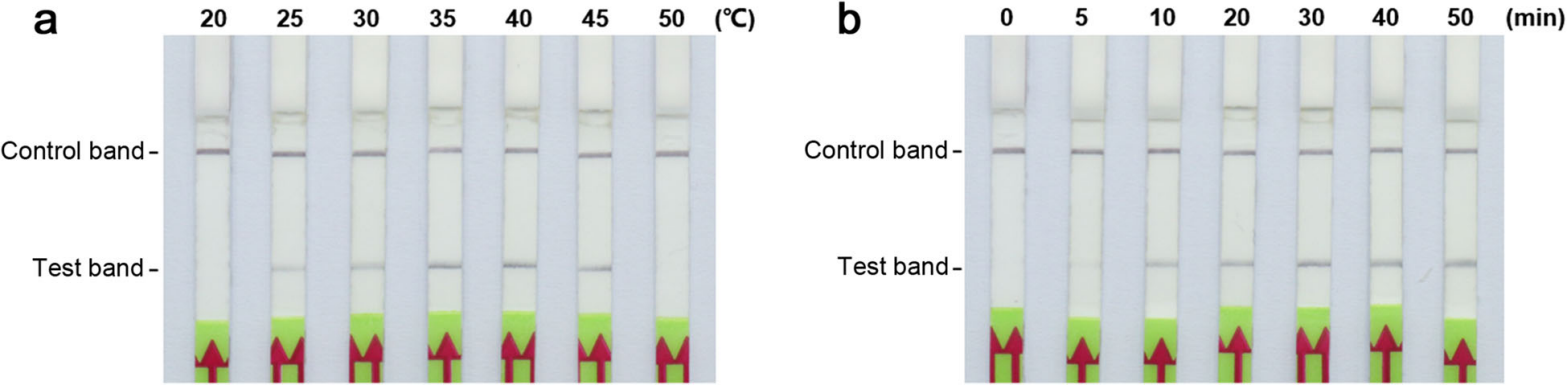
Optimization of a lateral flow recombinase polymerase amplification (LF-RPA) assay for the detection of *Phytophthora cactorum*. **a** Optimization of the RPA amplification temperature (top). **b** Evaluation of the RPA reaction time (top)

We determined the optimal temperature for RPA reactions using 1 ng of *P. cactorum* genomic DNA as template, testing a wide range of temperatures from 20 °C to 50 °C. The results showed clear test bands at 25–45 °C on lateral flow strips (Fig. 1a). No differences among amplicons were observed following RPA at 35 °C, 40 °C, or 45 °C, according to lateral flow assays (Fig. 1a). According to the manufacturer’s instructions and the stability of the reaction, we selected 39 °C as the optimal assay temperature. To determine the optimal reaction time, LF-RPA reactions were performed at 39 °C for durations ranging from 0 to 50 min, using 1 ng of *P. cactorum* genomic DNA as template. Amplification was conducted for 10 min and a faint test band was observed, followed by a clear positive test band at 20–50 min (Fig. 1b). The test bands for amplification products at 20, 30, 40, and 50 min were similar (Fig. 1b); therefore, we selected an incubation time of 30 min as the optimal time, in consideration of practicability.

### Detection sensitivity of the LF-RPA assay

**Fig. 2:**
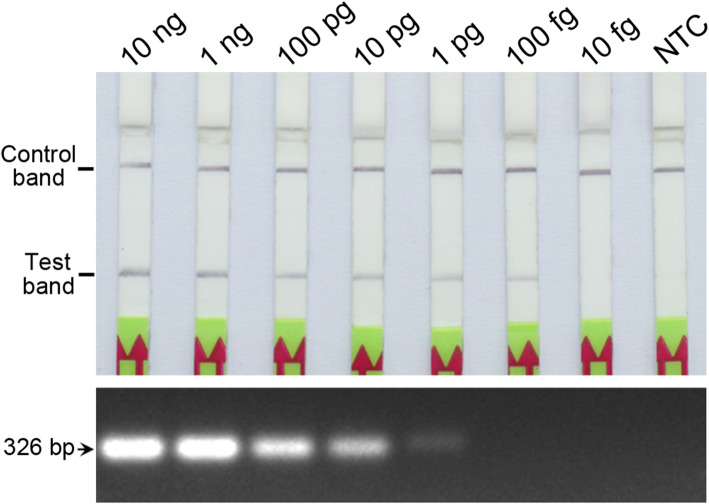
Comparison of the sensitivity of the LF-RPA assay (upper panel) and conventional polymerase chain reaction (PCR; lower panel) for *P. cactorum* detection. Serial dilutions of *P. cactorum* genomic DNA were used to evaluate the detection limit. NTC, no-template control. At least three replicate tests were performed

We tested the sensitivity of the LF-RPA assay using 10-fold serial dilutions of *P. cactorum* genomic DNA, and obtained a detection limit of 100 fg of genomic DNA (Fig. 2), which was at least 100 times greater than the detection limit of 1 pg for a conventional PCR assay (Fig. 2), indicating that the LF-RPA assay is more sensitive than conventional PCR in the detection of *P. cactorum* genomic DNA.

### Equipment-free detection of infected samples

**Fig. 3:**
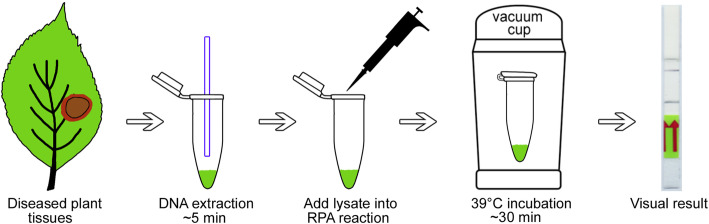
Workflow of the entire equipment-free detection process, including simplified DNA extraction and the LF-RPA assay

The LF-RPA assay was developed to detect *P. cactorum* in the field or in resource-limited laboratories. The general workflow for this process is illustrated in Fig. 3. The procedure for processing plant samples with the alkaline polyethylene glycol (PEG) reagent is extremely simple, allowing the extraction of total DNA within 5 min. Next, the specific primers and probe along with DNA template are introduced into the RPA reaction, followed by incubation for 30 min in a heat block at 39 °C. The entire RPA reaction process can be performed without any specialized equipment. After incubation, the amplicons are visualized directly using a lateral flow strip, on which the generated bands are read with the naked eye. Thus, the combination of simplified DNA extraction and the LF-RPA assay detects *P. cactorum* directly in field samples, and the results are obtained within approximately 40 min.

**Fig. 4:**
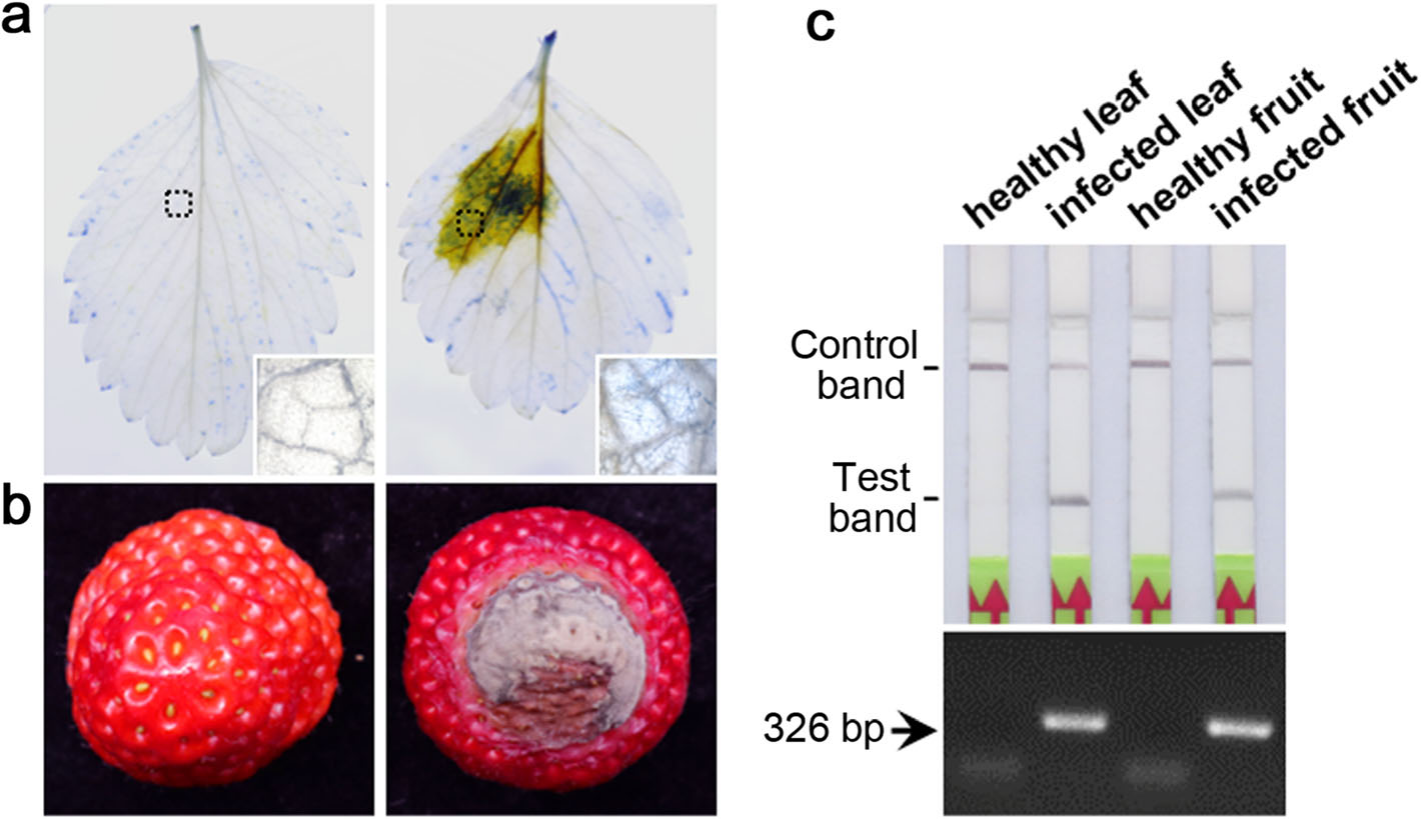
Evaluation of LF-RPA detection of *P. cactorum* in infected plant tissues. **a** *P. cactorum*-inoculated leaves at 5 days post-inoculation (dpi). Mycelia were visualized using trypan blue staining. **b** Representative disease symptoms of Phytophthora fruit rot in strawberry caused by *P. cactorum*. **c** LF-RPA and conventional PCR assays of genomic DNA extracted from healthy and infected strawberry tissues

To evaluate the efficacy of this method, we performed alkaline lysis extraction of plant genomic DNA and the LF-RPA assay after artificial infection of strawberry leaves. Strawberry leaves were inoculated with V8 agar containing actively growing *P. cactorum* mycelia for 5 days; mycelia in the infected plant leaves were observed through trypan blue staining (Fig. 4a). The LF-RPA assay showed positive results for the infected leaves, whereas no target band was observed in the negative control (Fig. 4c). Conventional PCR was used to further confirm the LF-RPA results (Fig. 4c). A total of 36 naturally infected strawberry fruits were tested using the LF-RPA assay, and 14 samples produced positive results (Fig. 4b, c). All 14 samples were also tested using a conventional PCR assay, and similar results were obtained. Therefore, the remaining 22 samples may have been infected by pathogens other than *P. cactorum*. Although the LF-RPA and PCR detection results were consistent, the LF-RPA assay was easier and faster to operate than conventional PCR.

## Discussion

The design of species-specific primers is critical for *P. cactorum* detection. The nucleotide sequence of the *Ypt1* gene varies sufficiently among *Phytophthora* species, and therefore has been widely used as a target for molecular detection of *Phytophthora* pathogens (Konig et al. 2015; Dai et al. 2019b; Yu et al. 2019; Lu et al. 2020). In this study, we also designed specific LF-RPA primers based on the *Ypt1* gene for *P. cactorum* detection. The specificity test showed that the designed Pcac-F3/R3 primer set distinguished *P. cactorum* from *P. infestans*, *P. ipomoeae*, *P. mirabilis*, and *P. parasitica*, all of which were clustered in *Phytophthora* phylogenetic clade 1. However, the *Ypt1* sequences of *P. cactorum* and closely related *Phytophthora hedraiandra* and *Phytophthora idaei* were very similar; therefore, it may be possible to amplify the same target DNA fragment from *P. hedraiandra* and *P. idaei* using the Pcac-F3/R3 primer set. Considering that our purpose was to detect *P. cactorum* in strawberry, and that *P. hedraiandra* and *P. idaei* have not been previously reported as strawberry pathogens, cross-reactivity between *P. cactorum* and *P. hedraiandra* or *P. idaei* will not affect the detection of *P. cactorum* in natural strawberry plants.

Thus, we developed an LF-RPA assay requiring no thermal cycling instruments that detects *P. cactorum* from infected strawberry samples within 40 min. The LF-RPA method has more advantages than other isothermal amplification methods, including simple primer design, high reaction efficiency, easy operation, and shorter operation time. The RPA system consists of recombinase single-strand DNA binding proteins and distinguishes DNA polymerase and amplifies DNA/RNA at a constant temperature without a thermal cycler. Although the RPA assay has many advantages among isothermal amplification methods, the cost of reaction reagents and lateral flow strips can be an obstacle in the development of field detection kits.

We detected the RPA amplification products using immunocolloidal gold test strips. Because the working principle of the RPA test is immunoprecipitation, its main cost is associated with coupled antibodies in the test strips. One way to reduce these costs is to reduce the number of combined monoclonal antibodies on the strips. In addition, microfluidic chips can be used to replace lateral flow strips for simultaneous detection of several pathogens, which would provide a new method for designing portable diagnostic kits. Such microfluidic chips have been applied for the successful bulk detection of plant viruses.

Compared with traditional molecular detection technologies, the LF-RPA assay is faster and more convenient. However, false positive signals sometimes appear in the negative control (Aebischer et al. 2014; Kim et al. 2018). Therefore, the steps of the LF-RPA process require further optimization. In the lateral flow strip detection step, the RPA reaction tubes must be opened to transfer the amplification product, introducing the risk of aerosol contamination and leading to cross-contamination. Therefore, the procedures for preparation and detection should be conducted in different dedicated rooms, in clean environments, opening and closing the reaction tubes carefully, and changing gloves as needed during detection. Recently, a microfluidic analytical system was successfully combined with LF-RPA for rapid and sensitive detection of SARS-CoV-2; this innovation greatly reduced the risk of exposure to aerosol contaminants (Liu et al. 2021), and is expected to be used for the rapid detection of plant pathogens in the near future.

## Conclusions

In summary, we developed a novel LF-RPA assay for the rapid and simple detection of *P. cactorum*. Including a simplified DNA extraction method, the entire detection process can be completed within 40 min, without the use of any specialized equipment. Thus, the LF-RPA assay has the potential to be developed into a portable detection kit for field detection of *P. cactorum* or other plant pathogens, particularly in resource-limited circumstances.

## Methods

### Pathogen cultivation

*P. cactorum* isolates were available in our laboratory. All *Phytophthora* and *Pythium* isolates were cultured on 10% vegetable juice (V8) agar medium at 25 °C in the dark. Pure cultures of different fungal strains were routinely maintained on potato dextrose agar (PDA) medium at 25 °C in the dark.

### DNA extraction and concentration detection

Pathogen mycelia were grown on 10% V8 agar or PDA medium at 25 °C for 3–5 days, then mycelia were harvested into 2-mL sterile centrifuge tubes and freeze-dried. Then, genomic DNA was extracted using the DNAsecure Plant Kit (DP320; Tiangen, Beijing, China), following the manufacturer’s instructions. The DNA was quantified by using the Qubit 3 Fluorometer and Qubit 1× dsDNA HS Assay Kit (Thermo Fisher Scientific, Wilmington, DE, USA). All samples were lysed in sterilized double-distilled H_2_O and stored at − 20 °C until use to avoid repeated freezing and thawing.

### Primer and probe design

We applied the widely used target gene *Ypt1* (ras-related GTP-binding protein 1 gene) to detect *P. cactorum*; *Ypt1* contains sufficient variation in non-coding regions and is suitable as molecular marker for nearly all *Phytophthora* spp. (Li et al. 2011, 2013). The *Ypt1* sequences of *P. cactorum* and closely related *Phytophthora* spp. were obtained from GenBank. Multiplex sequence alignment analysis was performed to search for specific regions of *Ypt1* in *P. cactorum*. Three RPA primer pairs were designed manually following instructions provided in the RPA guidelines (Twist Amp DNA Amplification Kits Combined Instruction Manual; TwistDx Ltd., Cambridge, UK). The likely secondary structures were assessed using the Multiple Primer Analyzer online tool to avoid hairpins, homodimers, heterodimers, and false priming. The length of each designed RPA primer was 33 or 36 bp, and the amplification product lengths ranged from 147 to 326 bp (Additional file 1: Table S1). To visualize the results using lateral flow detection, biotin was introduced into the sequence at the 5′ end of the reverse primer. A specific probe was designed exhibiting a 5′ fluorescein amidite, an abasic furan (dSpacer), and a 3′ C3 spacer (SpC3) (Additional file 1: Table S1). The primers and probes were synthesized by Sangon Biotech Co. (Shanghai, China).

Conventional PCR was initially used to evaluate the three primer pairs based on sensitivity and specificity detection. Each 25-μL reaction mixture consisted of 40 ng template DNA, 10 μM of each primer, and 2× *Taq* Master Mix (Vazyme Biotech, Nanjing, China). All reactions were completed in the SimpliAmp Thermal Cycler (A24812; Thermo Fisher Scientific) under the following conditions: initial denaturation at 95 °C for 3 min; 32 cycles of denaturation at 95 °C for 15 s, annealing at 60 °C for 15 s, and extension at 72 °C for 15 s; followed by a final extension at 72 °C for 5 min. The sensitivity assay was performed using 10-fold serial dilutions of *P. cactorum* genomic DNA, ranging from 1 ng to 100 fg. The PCR amplicons were visualized via agarose gel electrophoresis.

### Development of the LF-RPA assay to detect *P. cactorum*

The LF-RPA assay was performed using the TwistAmp nfo kit (TwistDx Ltd.) following the manufacturer’s instructions. The reaction mix consisted of an optimal amount of template DNA, 2.1 μM of each primer, 0.6 μM of target-specific lateral flow probe, nuclease-free water, and rehydration buffer (supplied in the kit). The solution was mixed by vortexing. We added the reaction mix to the freeze-dried reaction and mixed by pipetting. Then, 2.5 μL of magnesium acetate (supplied in the kit) was added to each well to start the reaction. The reaction was performed at 39 °C in a water bath for 30 min. After the mixtures had been incubated for 4 min, the reaction was paused; the samples were inverted 8–10 times for mixing, and then returned to the water bath. The RPA products were diluted 1:20 with HybriDetect assay buffer (supplied in the kit) in a sterile tube for analysis using lateral flow strips. The flow test strips were placed vertically in the reaction buffer mixture at room temperature for 2 min. Thus, the control line was visible regardless of the presence of the target line. The generation of a signal on the test line suggested that an antibody on the test line had captured its tag-carrying target, indicating a positive result. If a signal was generated only on the control line, then it was considered negative. The strips were placed on an A4 paper and photographed using a PowerShot SX720 HS camera (Canon, Tokyo, Japan).

### Optimization of LF-RPA conditions

To determine the optimal RPA reaction temperature, 1 ng of *P. cactorum* genomic DNA was used as a template to perform the assay. The reaction system was incubated at seven different temperatures (20 °C, 25 °C, 30 °C, 35 °C, 40 °C, 45 °C, and 50 °C) for 30 min. To determine the best detection time for the LF-RPA assay, amplification times of 0, 5, 10, 20, 30, 40, and 50 min were tested separately at 39 °C, using 1 ng of genomic DNA as template. The amplicons were immediately subjected to lateral flow strip detection.

### Evaluation of LF-RPA assay sensitivity

The sensitivity of the LF-RPA assay was evaluated by detecting serial dilutions of *P. cactorum* genomic DNA. Nuclease-free water was used instead of DNA as a no-template control (NTC). The reaction was performed at 39 °C for 30 min, and the amplification products were further analyzed using lateral flow strips. All tests were performed in triplicate under the same conditions.

### Equipment-free *P. cactorum* detection in infected strawberry samples

We evaluated the probability of detecting *P. cactorum* from artificially infected strawberry tissues. Isolated strawberry leaves were surface-sterilized with ethanol, and a 5-mm plug of V8 agar containing actively growing *P. cactorum* mycelia was placed face-down on the abaxial surface of each leaf. A 5-mm V8 agar plug without mycelia was used for inoculation as a negative control. The inoculated leaves were incubated in the dark at 25 °C for 5 days.

The *P. cactorum*-inoculated leaves were visualized using trypan blue staining, and DNA from two sample replicates was extracted using the quick alkaline lysis extraction method (Chomczynski and Rymaszewski 2006), with modifications. First, the leaves were cut into pieces (3 × 3 mm) and ground in a 1.5-mL tube using a pestle. The crushed pieces were lysed in 50 μL of reagent consisting of 6% PEG 200 (Sigma-Aldrich, Gillingham, UK) with 0.08% NaOH. The tubes were shaken by hand for 2 min at room temperature, and then left standing for 1 min. This simplified DNA extraction method can be performed within 5 min. We added 2 μL of the lysate to 50 μL of the LF-RPA mixture. Conventional PCR was also used to amplify the genomic DNA of all samples to verify the LF-RPA results.

To evaluate the effectiveness of the LF-RPA method in detecting *P. cactorum* in field samples, we collected 36 naturally infected strawberry fruits from Lishui and Jiangning, Nanjing, China, for LF-RPA detection. Total DNA was extracted following the alkaline lysis procedure described above, and the LF-RPA reactions were performed at 39 °C for 30 min in a water bath. The amplified products were observed using a lateral flow dipstick. Conventional PCR was used to amplify the genomic DNA of all samples to verify the LF-RPA results.

## Supplementary Information

**Additional file 1: Table S1.** Recombinase polymerase amplification (RPA) primers and the probe used in this study.

**Additional file 2: Figure S1.** Determination of the optimal primer combination for conventional polymerase chain reaction (PCR). **a** Sensitivity assay using 10-fold serial dilution of purified genomic DNA of *Phytophthora cactorum*. Agarose gel electrophoresis (2%) analysis of the PCR products. At least three repetitive tests were performed. **b** Specificity assay based on agarose gel electrophoresis. Lanes 1–10: 10 *P. cactorum* isolates collected from different geographic areas; Lanes 11–28: 18 isolates from *P. infestans*, *P. ipomoeae*, *P. mirabilis*, *P. parasitica*, *P. palmivora*, *P. capsica*, *P. sojae*, *P. megasperma*, *P. cryptogea*, and *P. drechsleri*; Lane 29–44: *Pythium spinosum*, *Py. intermedium*, *Py. helicoides*, *Py. ultimum*, *Py. aphanidermaum*, *Py. irregulare*, *Py. arrhenomanes*, *Py. hydnosporum*, *Py. marsipium*, *Py. dissotocum*, *Py. catenulatum*, *Py. splendens*, *Py. heterothallicum*, *Py. sylvaticum*, *Py. oligandrum* and *Py. periplocum*; Lane 45–48: *Fusarium graminearum*, *Sclerotinia sclerotiorum*, *Botrytis cinerea* and *Rhizopus oryzae*. At least three repetitive tests were performed.

## Data Availability

Not applicable.

## Abbreviations

ITS: Internal transcribed sequence
LAMP: Loop-mediated isothermal amplification
LF-RPA: Lateral flow recombinase polymerase amplification
PCR: Polymerase chain reaction
RPA: Recombinase polymerase amplification
