# Supplementary material for: Rapid and simple detection of Phytophthora cactorum in strawberry using a coupled recombinase polymerase amplification–lateral flow strip assay
Source: Phytopathol Res. 2021 Jun 10;3(1):12. doi: 10.1186/s42483-021-00089-8 (PMC8189726; doi:10.1186/s42483-021-00089-8)
Supplement: Supplementary file 1 — Additional file 1: Table S1. Recombinase polymerase amplification (RPA) primers and the probe used in this study. [file 42483_2021_89_MOESM1_ESM.docx]

**Table S1** Recombinase polymerase amplification (RPA) primers and the probe used in this study

| **Name** | **Sequenxe (5’ -3’)** | **Target size**  **(bp)** |
| --- | --- | --- |
| Pcac-F1  Pcac-R1 | CTTTGCGAGCTCCAGATTTCCACCAGAATTGCTGA  GTTGAATAGGAAACACGCCACGTCTGCTGGAGG | 147 |
| Pcac-F2  Pcac-R2 | CCTCCAGCAGACGTGGCGTGTTTCCTATTCAACTA  GTACACACGTTGGACGCACCTATCGATCTCGTGC | 217 |
| Pcac-F3  Pcac-R3 | CCAGAATTGCTGACATTCTTTTTTGCGGTTGTGCCC  (Biotin-)TTCCGTCGGCTCTTTCAGTACACACGTTGGACG | 326 |
| PcacProb | FAM-TCCTATTCAACTAACGGTCCACTTGTGTGT-THF-TGTAGTGGGACACGGCC-C3 space |  |
