# Supplementary material for: Rapid and simple detection of Phytophthora cactorum in strawberry using a coupled recombinase polymerase amplification–lateral flow strip assay
Source: Phytopathol Res. 2021 Jun 10;3(1):12. doi: 10.1186/s42483-021-00089-8 (PMC8189726; doi:10.1186/s42483-021-00089-8)
Supplement: Supplementary file 2 — Additional file 2: Figure S1. Determination of the optimal primer combination for conventional polymerase chain reaction (PCR). a Sensitivity assay using 10-fold serial dilution of purified genomic DNA of Phytophthora cactorum. Agarose gel electrophoresis (2%) analysis of the PCR products. At least three repetitive tests were performed. b Specificity assay based on agarose gel electrophoresis. Lanes 1–10: 10 P. cactorum isolates collected from different geographic areas; Lanes 11–28: 18 isolates from P. infestans, P. ipomoeae, P. mirabilis, P. parasitica, P. palmivora, P. capsica, P. sojae, P. megasperma, P. cryptogea, and P. drechsleri; Lane 29–44: Pythium spinosum, Py. intermedium, Py. helicoides, Py. ultimum, Py. aphanidermaum, Py. irregulare, Py. arrhenomanes, Py. hydnosporum, Py. marsipium, Py. dissotocum, Py. catenulatum, Py. splendens, Py. heterothallicum, Py. sylvaticum, Py. oligandrum and Py. periplocum; Lane 45–48: Fusarium graminearum, Sclerotinia sclerotiorum, Botrytis cinerea and Rhizopus oryzae. At least three repetitive tests were performed. [file 42483_2021_89_MOESM2_ESM.docx]

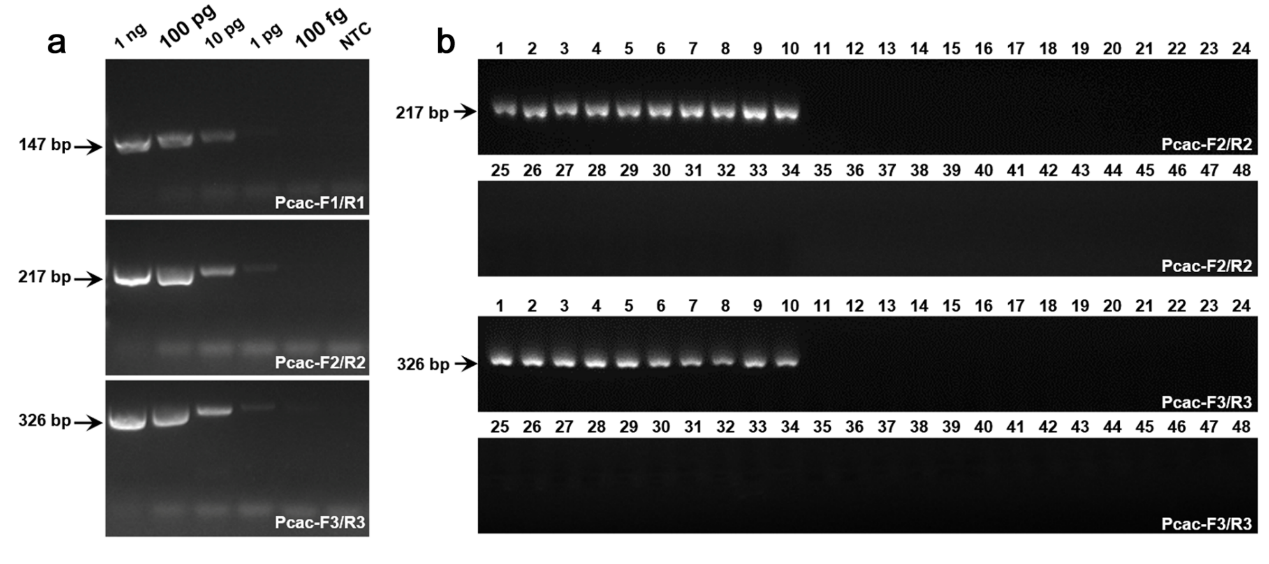


**Additional file 2: Figure S1.** Determination of the optimal primer combination for conventional polymerase chain reaction (PCR). **a** Sensitivity assay using 10-fold serial dilution of purified genomic DNA of *Phytophthora cactorum*. Agarose gel electrophoresis (2%) analysis of the PCR products. At least three repetitive tests were performed. **b** Specificity assay based on agarose gel electrophoresis. Lanes 1-10: 10 *P. cactorum* isolates collected from different geographic areas; Lanes 11-28: 18 isolates from *P. infestans*, *P. ipomoeae*, *P. mirabilis*, *P. parasitica*, *P. palmivora*, *P. capsica*, *P. sojae*, *P. megasperma*, *P. cryptogea*, and *P. drechsleri*; Lane 29-44: *Pythium spinosum*, *Py. intermedium*, *Py. helicoides*, *Py. ultimum*, *Py. aphanidermaum*, *Py. irregulare*, *Py. arrhenomanes*, *Py. hydnosporum*, *Py. marsipium*, *Py. dissotocum*, *Py. catenulatum*, *Py. splendens*, *Py. heterothallicum*, *Py. sylvaticum*, *Py. oligandrum* and *Py. periplocum*; Lane 45-48: *Fusarium graminearum*, *Sclerotinia sclerotiorum*, *Botrytis cinerea* and *Rhizopus oryzae*. At least three repetitive tests were performed.
